# Wood stove use and other determinants of personal and indoor exposures to particulate air pollution and ozone among elderly persons in a Northern Suburb

**DOI:** 10.1111/ina.12538

**Published:** 2019-02-20

**Authors:** Taina Siponen, Tarja Yli‐Tuomi, Pekka Tiittanen, Pekka Taimisto, Juha Pekkanen, Raimo O. Salonen, Timo Lanki

**Affiliations:** ^1^ Environmental Health Unit, Department of Health Security National Institute for Health and Welfare Kuopio Finland; ^2^ Department of Public Health University of Helsinki Helsinki Finland; ^3^ School of Medicine University of Eastern Finland Kuopio Finland; ^4^ Department of Environmental and Biological Sciences University of Eastern Finland Kuopio Finland

**Keywords:** black carbon, building ventilation, candle burning, cooking, fine particulate matter, wood combustion

## Abstract

A six‐month winter‐spring study was conducted in a suburb of the northern European city of Kuopio, Finland, to identify and quantify factors determining daily personal exposure and home indoor levels of fine particulate matter (PM_2.5_, diameter <2.5 µm) and its light absorption coefficient (PM_2.5abs_), a proxy for combustion‐derived black carbon. Moreover, determinants of home indoor ozone (O_3_) concentration were examined. Local central site outdoor, home indoor, and personal daily levels of pollutants were monitored in this suburb among 37 elderly residents. Outdoor concentrations of the pollutants were significant determinants of their levels in home indoor air and personal exposures. Natural ventilation in the detached and row houses increased personal exposure to PM_2.5_, but not to PM_2.5abs_, when compared with mechanical ventilation. Only cooking out of the recorded household activities increased indoor PM_2.5_. The use of a wood stove room heater or wood‐fired sauna stove was associated with elevated concentrations of personal PM_2.5_ and PM_2.5abs_, and indoor PM_2.5abs_. Candle burning increased daily indoor and personal PM_2.5abs_, and it was also a determinant of indoor ozone level. In conclusion, relatively short‐lasting wood and candle burning of a few hours increased residents’ daily exposure to potentially hazardous, combustion‐derived carbonaceous particulate matter.

**Practical Implications**

Our study adds as the most important message to the present scientific and public health information that ample burning of wood in small‐scale room heaters and sauna stoves is likely to increase chronic personal exposures in the neighborhood to particulate matter that contains substantial amounts of soot and hazardous organic compounds like polycyclic organic hydrocarbons. This exposure does not take place only while staying outdoors but also indoors at home due to effective passage of the small particles through the building shield. Part of the emissions adding this type of hazardous exposure among residents, also including susceptible population groups, originates directly from the personal use of a wood‐fired room heater or sauna stove. Insufficient natural ventilation in older houses further elevates the indoor levels of the hazardous particles.

## 1 INTRODUCTION

Exposure to ambient air particulate matter (PM) has been found to be detrimental to health. For example, associations between PM and increased cardiovascular and respiratory mortality and morbidity have been established.^1^, ^2^, ^3^ The elderly, young children and persons with lower economic status or chronic cardiorespiratory disease are the most susceptible to the harmful influences of air pollution.^3^ Exposure to fine particulate matter (PM_2.5_; aerodynamic diameter <2.5 µm) is regarded as the most important environmental risk factor when assessing the burden of disease.^4^, ^5^ Many factors affect the indoor and personal exposures and possibly the risks of adverse health effects in affected population. Such factors are, for example, outdoor PM mass concentration, size distribution, and chemical composition, as well as building characteristics.^1^, ^6^, ^7^

The systematic review of Janssen et al^8^ stated that black carbon is an important health‐relevant component of PM. However, the authors concluded that the associated health effects are likely not due to combustion‐derived solid black carbon particles per se, but rather due to harmful organic and inorganic constituents adsorbed in it during cooling and gas‐to‐liquid transition of the emissions and in subsequent physicochemical processes in the atmosphere. Black carbon and numerous organic carbon compounds are formed in incomplete combustion of fossil fuels in vehicle engines and power plants as well as biomass in small‐scale residential heaters. Thus, black carbon can be used as a universal indicator of combustion‐derived particles in PM‐related exposure and health studies. In fact, detailed analysis of the chemical composition of PM is laborious and expensive, and the present scientific knowledge is highly insufficient, especially regarding the harmfulness of organic compounds other than polycyclic aromatic hydrocarbons (PAH). The reflectance of filters, transformed into absorption coefficient, can be used as an estimate of black carbon.^9^

High concentrations of ozone (O_3_) close to earth's surface have harmful effects on health. The majority of tropospheric ozone near urban areas is formed as a secondary pollutant in a complex reaction chain where nitrogen oxides (NO_x_), volatile organic compounds (VOCs), and sunlight are the key actors.^10^ In this study area, traffic exhaust emissions and residential wood combustion were the main sources of precursors of tropospheric ozone. As ozone formation requires sunlight for completion, outdoor ozone concentration is low in winter, especially in high latitudes, and increases considerably in spring.^11^ The concentrations are usually somewhat lower in city centers compared to suburban and rural areas, because ozone reacts with NO originating as the primary nitrous emission product from tailpipes.^11^ In short‐term health studies, ozone has been associated with both respiratory and cardiovascular health outcomes including mortality and hospital admissions,^6^, ^12^, ^13^ and also with out‐of‐hospital cardiac arrest, according to the systematic review and meta‐analysis conducted by Zhao et al.^14^ However, in a recent quantitative review of Atkinson et al,^15^ annual outdoor ozone levels were not systematically associated with morbidity. Generally, there is a lack of information on ozone levels and its determinants in home indoor environments in the northern subarctic climate.

In most short‐term studies, exposure assessment is based on air pollution measurements at one or few central sites. However, central site measurements may not effectively illustrate home outdoor concentrations, especially in areas with intense low‐height emissions from local sources such as residential wood combustion or traffic emissions in major roads.^16^, ^17^, ^18^, ^19^ Corresponding weak relationships have also been found between outdoor and personal exposure concentrations of PM_2.5_.^20^, ^21^ Despite significant positive correlations observed between indoor and outdoor ozone concentrations,^22^, ^23^ the indoor ozone levels have been low compared to the outdoor levels and are strongly influenced by the building ventilation and airtightness of its shield. Since people spend most of their daily time indoors in developed countries, their total personal exposure depends mostly on indoor concentrations of air pollutants, in other words on infiltrated outdoor air pollutants and indoor‐generated pollutants.

In the review article of Morawska et al,^24^ it has been assessed that 10%‐30% of the total burden of disease from particulate matter exposure in developed countries resulted from indoor‐generated particles. However, there is far less information available on exposures to indoor‐generated PM_2.5_ than there is on outdoor PM_2.5_. Smoking and cooking have been suggested to be the main sources of indoor air particles at home.^25^, ^26^, ^27^, ^28^, ^29^ Other possible indoor sources of air pollution are the use of personal care products,^30^, ^31^ candle burning,^26^, ^29^ wood burning,^21^ home cleaning,^28^ pets,^30^ and gypsum/wallboard.^31^ Regardless, in developed countries, there are still a limited number of studies where personal and indoor determinants of wood burning are studied. And those already reported are in principally in areas with high concentrations.^21^

The objective of this study was to identify and quantify factors determining personal exposure and indoor levels of PM_2.5_ and its light absorption coefficient (PM_2.5abs_) as well as indoor ozone concentration in houses located in a suburb of a Northern European city that has low annual air pollution levels. Our special interest was to evaluate the relevance of wood combustion as an exposure determinant in a suburb where wood is mainly burned in secondary heating devices and in sauna stoves.

## 2 MATERIALS AND METHODS

### 2.1 Study design

The study was conducted in a suburb called Jynkkä in the City of Kuopio, Central Eastern Finland, during the heating season between November 2008 and May 2009. At that time, Kuopio had about 98 000 inhabitants. Around 3400 inhabitants lived in this low population density suburb, which is located 6‐8 km south of the city center of Kuopio. Nearly 100% of the houses in this suburb had district heating as their primary heating source.

A central air pollution measurement site was set up to determine air pollutant concentrations prevailing in the study area. The sample intakes were about 5 m above ground level. Sampling inlets were placed 1.5 m above the roof of the container (container roof was about 3.5 m above the ground) in order to allow free air movements around the inlets and prevent vandalism. The nearest relatively busy road (traffic intensity >5000 vehicles per 24 hours) was at a distance of over 1 km. Estimate of traffic intensity was based on a traffic census made by the City of Kuopio, and the distance was determined using ArcMap. A small oil‐fired (low sulfur fuel oil) district heating plant belonging to the reserve capacity in the network was located 300 m south‐southwest of the central measurement site. It operated 0‐118 hours (average 43 hours) per month. Effects of emissions from this heating plant could not be detected from the time series data of pollutant concentrations (data not shown).

Detailed results from the central site outdoor air quality measurements have been previously described by Yli‐Tuomi et al.^19^ Fine particle sources that were identified in the study area are long/regional‐range transport, tail‐pipe emissions, indirect traffic emissions (road dust), sea spray, and wood combustion. Mean levels of outdoor PM_2.5_ and ozone measured in the central air pollution measurement site were 4.8 and 55 µg/m^3^ between November 10, 2008 and May 19, 2009.

To determine daily personal exposure to PM_2.5_ and PM_2.5abs_, each participant in the panel of 37 elderly study subjects carried a backpack containing a personal measurement system for 22 hours each month. Indoor measurements of PM_2.5_, PM_2.5abs_, and ozone were conducted at the same time with personal measurements in the homes of the study subjects. The median distance between the central site and home location was 0.54 km. Samples were collected at a height of 1 m in the living room or other common space used by all residents of the house. Sampling height of 1 m represents well a person sitting in a chair or sleeping in a bed. Each person had four to six repeated 22‐hour measurements during the 6‐month field campaign. Measurements at homes of study subjects lasted only 22 hours (24 hours at central site), as same devices were transported from one home to another between the measurement days. Although indoor and personal measurements were done at the same time, the total amount of them may differ, as some measurements failed because of equipment breakage, subjects switching off equipment (noise at nighttime), and appointment cancellation. Outdoor measurements were done continuously (except for a short Christmas break) during the whole study period.

Information on housing characteristics as well as on subjects’ activities, potentially affecting personal exposure or indoor pollutant concentrations, was collected with questionnaires and time‐activity diaries. Baseline questionnaires filled in by the researchers were used to collect information on constant variables during the study period, such as gender, education, home type, ventilation system, and distance between home and collector road. These are called “time‐invariant determinants” in this study. Type of air exchange where building air supply and extraction are based on pressure differences inside and outside the building is referred as natural ventilation system. Ventilation is referred to as mechanical when air extraction or both air supply and extraction are carried out using a fan or other mechanical system. These types of ventilation systems are typical for the homes in this study, as they were built mainly in the seventies and eighties (building years: 1976‐1987, 2000). A self‐administrated questionnaire, filled in during each 22‐hour measurement period, was used to collect information on potential “time‐varying determinants”, such as cooking, use of a wood stove (room heater) or sauna stove, smelling of wood smoke odor, and time spent in different microenvironments. Time‐activity diaries were filled in with 15‐minute resolution.

### 2.2 Sampling and sample analysis

Sampling methods have been described in more detail in the paper of Yli‐Tuomi et al.^19^ Briefly, PM_2.5_ samples were collected by using sampling system containing in series a PM_2.5_ cyclone (GK 2.05 KTL; BGI, Waltham, MA, USA), a data logging photometer (pDR‐1200 X; MIE, Bedford, MA, USA), a filter holder (M000037A0; Millipore, Bedford, MA, USA), and an air pump (AFC400S; BGI). Samples were collected on 37 mm, 2‐µm pore size Pall Life Sciences Zefluor membrane filters (Pall Corporation, Ann Arbor, MI, USA) with Nucleopore Drain Disc (Whatman International Ltd., Ghent, UK) as support. The sampler operated with a volume flow rate of 4 L/min. Personal and home indoor measurements were scheduled to start between 10 am and 12 noon and end between 8 and 9 am the following morning. At the central outdoor measurement site, PM filters were changed daily at 9 am. Zefluor membrane filters were weighed before and after sampling by using a Mettler M3 microbalance (Mettler Instumente, Zurich, Switzerland) with 1 µg reading resolution. The filters were stabilized in a weighing room for about 24 hours before weighing. A standard weight was weighed before and after each session to verify the scale reliability. Po‐210 radioactive source (1U400 static master; NRD, Grand Island, NY, USA) was used to remove static electricity. After weighing, the samples were stored in a cold room (+5°C) until the reflectance measurements were made.

PM_2.5abs_, an indicator of elemental or black carbon and thus combustion‐derived carbonaceous particles, was determined by measuring reflectance of the PM_2.5_ filters with a smoke‐stain reflectometer (Model M43D; Diffusion systems, London, UK). Reflectance of the PM_2.5_ filter was transformed into an absorption coefficient (*a*) according to ISO9835^32^:

(1) $$a\;=\;\left(\frac{A}{2V}\right)\cdot \ln\;\left(\frac{R_{0}}{R_{\text{s}}}\right)$$

where *A* = loaded filter area (m^2^), *V* = sampled volume (m^3^), *R*_0_ = average reflectance of field blank filters, and *R*_s_ = reflectance of the sampled filter. The light absorption coefficient (PM_2.5abs_) is expressed as m^−1^ × 10^−5^.

Ozone in the home indoor air was measured continuously with a photometric O_3_ analyzer (Model 400E; Teledyne Instruments, Advanced Pollution Instrumentation Division, San Diego, CA, USA), while another photometer (Environment O3 42M Environnement S.A, Poissy Cedex, France) was used at the central outdoor site. The home indoor monitors were factory calibrated, while the monitor located at the central site station was calibrated in the accredited laboratory of the Finnish Meteorological Institute. The 22‐hour average ozone concentration during each home indoor measurement was calculated from the continuous data to match the indoor PM sampling period. At the central outdoor measurement site, ozone concentrations were calculated from 9 am to 9 am the next day.

### 2.3 Statistical analyses

The strength of the relationships between daily personal, home indoor, and central site outdoor measurement data was examined by calculating subject‐specific Spearman correlation coefficients. The distribution of subject‐specific coefficients was represented by quartiles (25th, 50th, and 75th percentiles). Statistical analyses were performed using linear mixed models with random subject effects in SAS statistical software version 9.3. Based on the distribution of studentized residuals, personal and indoor measurements of PM and O_3_ were log‐transformed to remove skewness and to improve the normal distribution of the data before statistical analysis. If a subject had fewer than four valid observations out of a maximum of six, all measurements of the pollutant in question were excluded. Measurements were invalid if measurement time was short (equipment malfunction or subjects shutdown the devices because of noise) or measurements were canceled (eg, subject had schedule problems or flu). Each pollutant was analyzed separately, and thus, less than four valid O_3_ measurements did not lead to exclusion of this person from the PM analysis. Time‐invariant and time‐varying determinants were included in the model to test whether they predicted personal exposure or home indoor pollutant concentration. Model building was done in two steps. First, only one potential determinant at a time was included in a simple model adjusted for outdoor air levels of the pollutant. Secondly, all determinants having *P*‐value less than 0.25 at the first step were included in the final model adjusted for outdoor air pollutant levels. Associations are presented as percentage changes in personal PM, indoor PM, and indoor O_3_ concentration.

As a sensitivity analysis, measurements having studentized residual over 2 or 3 were removed separately. Furthermore, a definition of cooking was changed by replacing the determinant representing “cooking with a frying pan or in an oven” with a general cooking variable including all cooking activities except for the use of a microwave or coffee machine. An additional sensitivity analysis was performed by replacing the variable “use of wood stove—burning wood” with “wood smoke odor” describing the frequency of odor observations indoors or outdoors. PM_2.5_ levels from wood burning, determined in the previous study by source apportionment of the outdoor PM_2.5_ concentrations measured at the central site monitoring station,^19^ were also tested in the final models.

## 3 RESULTS

In this study, elderly study subjects spent on average 92% (89%) of their time indoors (at home indoors). Descriptive statistics of the personal exposure, home indoor, and central site outdoor pollutant concentrations are presented in Table 1. The daily median values of the personal, home indoor, and central site outdoor PM_2.5_ were 3.3, 3.9, and 4.2 µg/m^3^, respectively. For indoor and outdoor ozone, the daily median levels were 4.2 and 55.2 µg/m^3^, respectively. The PM_2.5_ concentrations were mainly low, but the levels on a few measurement days were more than 15 times higher (maximums 55.4 µg/m^3^ for personal PM_2.5_ and 66.7 µg/m^3^ for indoor PM_2.5_) than the overall median values. Similar differences were also found in personal PM_2.5abs_. In indoor PM_2.5abs_ and ozone levels, the differences were relatively minimal.

**Table 1:** Descriptive statistics of daily levels of personal, home indoor, and central outdoor PM_2.5_, PM_2.5abs_, O_3_, and PM_2.5_ from wood burning

	n_s_	n	Mean	SD	25%	50%	75%	Max
PM_2.5_ [µg/m^3^]								
Personal	28	153	4.3	5.3	2.0	3.3	4.8	55.4
Home indoor	36	196	5.0	5.9	2.6	3.9	5.5	66.7
Central outdoor		113	4.8	3.7	2.2	4.2	6.2	15.3
PM_2.5abs_ [m^−1^ × 10^−5^]								
Personal	28	155	0.3	0.3	0.1	0.2	0.4	3.2
Home indoor	36	196	0.2	0.2	0.1	0.2	0.3	1.7
Central outdoor		114	0.3	0.3	0.1	0.3	0.5	1.0
O_3_ [µg/m^3^]								
Home indoor	27	134	6.2	6.3	2.6	4.2	7.8	41.3
Central outdoor		116	55.1	22.0	37.8	55.2	73.5	105
WOOD [µg/m^3^]								
Central outdoor		110	0.9	0.8	0.3	0.7	1.3	4.2

n, number of measurements; n_s_, number of subjects; O_3_, ozone; PM_2.5_, fine particulate matter (<2.5 µm in aerodynamic diameter); PM_2.5abs_, light absorption coefficient of PM_2.5_; SD, standard deviation; 25%‐75%, 25‐75th percentile; WOOD, central site PM_2.5_ concentration from wood burning.^19^

Median Spearman correlation coefficients between daily personal, home indoor, and central site outdoor levels of measured air pollutants for individual subjects are presented in Table 2. Correlations were generally low, and in general, interquartile ranges were wide. Personal exposure to PM_2.5_ had high correlations of 0.80 with both home indoor and central site outdoor PM_2.5_ concentrations. Central outdoor PM_2.5_ correlated relatively highly with central outdoor PM_2.5abs_ (*R* = 0.77).

**Table 2:** Medians (25th and 75th percentile) of Spearman correlation coefficients calculated for study subjects

	Personal		Home indoor			Central outdoor	
	PM_2.5_	PM_2.5abs_	PM_2.5_	PM_2.5abs_	O_3_	PM_2.5_	PM_2.5abs_
Personal							
PM_2.5abs_	0.55 (0.18, 0.8)						
Home indoor							
PM_2.5_	0.80 (0.3, 1.0)	0.50 (0.1, 0.77)					
PM_2.5abs_	0.40 (0.1, 0.70)	0.60 (0.4, 0.8)	0.54 (0.14, 0.77)				
O_3_	0.00 (−0.5, 0.4)	0.09 (−0.54, 0.45)	−0.20 (−0.4, 0.5)	−0.10 (−0.3, 0.6)			
Central outdoor							
PM_2.5_	0.80 (0.3, 0.94)	0.45 (−0.06, 0.76)	0.40 (0.2, 0.8)	0.31 (−0.06, 0.7)	0.00 (−0.4, 0.4)		
PM_2.5abs_	0.40 (0.1, 0.75)	0.50 (0.01, 0.8)	0.40 (0.2, 0.7)	0.52 (0.03, 0.74)	−0.20 (−0.5, 0.2)	0.77 (0.6, 0.94)	
O_3_	0.06 (−0.25, 0.4)	−0.25 (−0.6, 0.1)	−0.17 (−0.5, 0.3)	−0.28 (−0.6, 0.18)	0.60 (−0.1, 0.9)	0.14 (−0.3, 0.37)	−0.26 (−0.54, 0.09)

O_3_, ozone; PM_2.5_, fine particulate matter, less than 2.5 µm in aerodynamic diameter; PM_2.5abs_, light absorption coefficient of PM_2.5_.

Daily median levels of PM_2.5_, PM_2.5abs_, and ozone in the categories of time‐invariant determinants are shown in Table 3. Personal and indoor PM_2.5_ and PM_2.5abs_ levels were slightly higher for subjects living near a collector road (<50 m). The effect was opposite for ozone concentration. Indoor PM_2.5_ levels were higher among the subjects frequently reporting wood smoke odor at home indoors. In relative terms, the same trend also came up in indoor PM_2.5abs_ levels, but not as clearly in personal PM_2.5abs_.

**Table 3:** Daily median levels of PM_2.5_, PM_2.5abs_, and O_3_ by time‐invariant determinants of exposure

	Personal				Indoor					
	n_s_	PM_2.5_ [µg/m^3^]	n_s_	PM_2.5abs_ [m^−1^ × 10^−5^]	n_s_	PM_2.5_ [µg/m^3^]	n_s_	PM_2.5abs_ [m^−1^ × 10^−5^]	n_s_	O_3_ [µg/m^3^]
Ventilation										
Natural	12	3.6	12	0.16	12	4.2	12	0.19	6	3.2
Mechanical	20	3.2	20	0.21	24	3.7	24	0.18	21	4.3
Pets										
Yes	9	3.2	9	0.17	9	3.7	9	0.18	4	3.0
No	23	3.4	23	0.20	27	3.9	27	0.19	23	4.3
Collector road within 50 m										
Yes	8	3.6	8	0.22	10	4.3	10	0.23	5	3.7
No	24	3.1	24	0.19	26	3.7	26	0.18	22	4.5
Wood smoke odor at home indoors										
1‐4 d per week	6	3.2	6	0.21	7	4.7	7	0.22	6	2.9
<1 d per week	9	3.3	9	0.20	11	3.9	11	0.19	8	4.3
Never	17	3.4	17	0.19	18	3.7	18	0.17	13	0.7

n_s_, number of subjects; O_3_, ozone; PM_2.5_, fine particulate matter, less than 2.5 µm in aerodynamic diameter; PM_2.5abs_, light absorption coefficient of PM_2.5_.

Daily median levels of PM_2.5_, PM_2.5abs_, and ozone in the categories of time‐varying determinants are presented in Table 4. Indoor, but not personal PM_2.5_ levels were mostly higher, when nearly any indoor activity was done at home. Personal and indoor PM_2.5abs_ levels were consistently higher with determinants related to wood or candle burning.

**Table 4:** Daily median levels of PM_2.5_ (µg/m^3^), PM_2.5abs_ (m^−1^ × 10^−5^) and O_3_ (µg/m^3^) by time‐varying determinants of exposure

	Personal				Indoor					
	n^1^	PM_2.5_ [µg/m^3^]	n^1^	PM_2.5abs_ [m^−1^ × 10^−5^]	n^1^	PM_2.5_ [µg/m^3^]	n^1^	PM_2.5abs_ [m^−1^ × 10^−5^]	n^1^	O_3_ [µg/m^3^]
Window open										
≥1 h	41	3.3	41	0.17	56	4.4	56	0.16	39	4.3
<1 h	61	3.2	62	0.19	72	4.0	73	0.21	51	3.6
No	47	3.3	48	0.21	65	3.5	64	0.17	41	4.4
Candle burning										
Yes	19	2.4	19	0.27	28	4.2	29	0.26	15	4.8
No	133	3.3	135	0.19	168	3.8	167	0.18	118	4.1
Chemical use										
Yes	12	2.6	12	0.08	17	3.9	17	0.15	10	4.4
No	139	3.3	141	0.21	178	3.9	178	0.19	122	4.1
Wood smoke odor										
Yes	38	3.7	38	0.23	50	4.6	49	0.23	40	3.3
No	115	3.2	117	0.19	146	3.7	147	0.18	94	4.5
Cooking via frying or oven										
Yes	67	3.1	69	0.18	93	4.4	92	0.21	61	4.3
No	86	3.4	86	0.21	103	3.4	104	0.17	73	4.1
House cleaning										
Yes	37	3.6	38	0.18	44	4.5	43	0.21	33	4.3
No	116	3.2	117	0.20	152	3.8	153	0.18	101	4.1
Aerosol use										
Yes	19	2.6	19	0.23	22	3.9	22	0.22	19	3.8
No	134	3.4	136	0.20	174	3.9	174	0.18	115	4.3
Use of wood stove—burning wood										
Yes	39	3.2	39	0.24	52	4.1	52	0.19	43	3.0
No	114	3.3	116	0.19	144	3.9	144	0.18	91	4.8
Use of wood stove—feeding and flame management										
Yes	34	3.1	35	0.24	46	3.7	46	0.19	39	3.1
No	119	3.4	120	0.19	150	4.0	150	0.18	95	4.5
Time spent (h)										
In a motor vehicle										
0	81	3.3	81	0.15	95	3.7	96	0.20	68	4.1
1	33	3.8	33	0.20	41	4.5	41	0.19	28	3.7
≥2	39	3.1	41	0.25	60	3.8	59	0.16	38	4.6
Indoors elsewhere										
0	86	3.4	87	0.15	113	3.9	114	0.19	78	3.9
1‐3	28	2.8	28	0.21	33	3.6	33	0.18	20	6.3
≥4	39	3.5	40	0.27	50	4.1	49	0.20	36	4.7
Outdoors										
0	27	3.3	27	0.18	37	4.0	37	0.21	22	3.6
1‐2	21	3.6	22	0.27	29	4.7	28	0.20	20	4.4
≥3	105	3.3	106	0.20	130	3.8	131	0.18	92	4.3

n^1^, total number of measurements; O_3_,ozone; PM_2.5_, fine particulate matter, less than 2.5 µm in aerodynamic diameter; PM_2.5abs_, light absorption coefficient of PM_2.5_.

Determinants of the personal and indoor air pollutants included in the final models are shown in Table 5. Building ventilation (natural vs mechanical) was a powerful determinant in both the indoor and personal PM_2.5_ models. Aerosol use, for example, use of cosmetic or household cleaning products, and cooking were included in the indoor PM_2.5_ model, while the burning of wood was strongly involved in the personal PM_2.5_ model. Burning of wood and candle burning were powerful determinants in both PM_2.5abs_ models. In addition, closeness of the collector road, keeping a window open, time spent in a motor vehicle, spending time indoors elsewhere than home, and cooking turned out to be determinants of personal exposure to PM_2.5abs_. The proximity of a collector road, keeping a window open, candle burning, and burning of wood were parameters included in the model explaining indoor ozone concentrations.

**Table 5:** Percentage changes in PM_2.5_, PM_2.5abs_, and O_3_ by time‐invariant and time‐varying determinants in multi‐determinant models adjusted for outdoor levels

	Personal		Indoor		
	PM_2.5_	PM_2.5abs_	PM_2.5_	PM_2.5abs_	O_3_
Natural vs. mechanical ventilation	21**		19		
Collector road within 50 m		4			‐4
Window open ≥1 h		5			6
Candle burning		8*		10***	3**
Cooking with frying or oven		−2	15**		
Aerosol use			−14		
Use of wood stove—burning wood	20*	9**		7***	−2
Time spent in a motor vehicle ≥2 h		2			
Time spent indoors elsewhere ≥4 h		7	b	b	b
Outdoor PM_2.5_, PM_abs_, or O_3_ a	7***	21***	5***	3***	1***
Number of measurements	148	148	190	192	131

PM_2.5_, particles <2.5 µm in aerodynamic diameter, PM_2.5abs_, light absorption coefficient of PM_2.5_.

a Continuous variable, estimate calculated for a change of 1 µg/m^3^ in PM_2.5_ and O_3_ and 1 m^−1^ × 10^−5^ in PM_abs_.

b Determinant not relevant for indoor concentrations.

* *P* < 0.1.

** *P* < 0.05.

*** *P* < 0.01.

During the measurement days when the study subjects burned wood in their masonry heater, wood‐fired sauna stove or recreational open fireplace, burning lasted on average 2.3 h/d. Accordingly, candle burning lasted on average 3.6 h/d. These activities were associated with nearly the same magnitude increases in personal PM_2.5abs_: wood burning 9% and candle burning 8%.

In general, no substantial changes in the results were found in various sensitivity analyses of the multi‐determinant models. After the exclusion of high residuals (using either 2 or 3 as the cutoff value), the associations became slightly weaker, but the general pattern remained the same. After replacement of the original cooking variables (cooking with frying pan or in oven) with a more general cooking variable, including all cooking activities except for a using microwave or coffee machine, the association with indoor PM_2.5_ was no longer significant. Previously modeled outdoor PM_2.5_ concentration from wood burning in the study area^19^ was not a determinant of personal or indoor PM_2.5_ or PM_2.5abs_ concentration.

## 4 DISCUSSION

We identified and quantified factors determining personal exposures and home indoor levels of PM_2.5_ and PM_2.5abs_, as well as indoor ozone concentration in a suburb of a northern European city that has generally low ambient air pollution levels. We found outdoor concentrations to be statistically significant determinants of personal and indoor PM_2.5_ and PM_2.5abs_, and also indoor ozone. Furthermore, residential wood combustion and candle burning were significantly associated with both personal and indoor PM_2.5abs_ levels and wood combustion also with personal PM_2.5_. Single associations between personal or indoor air pollutants and building ventilation or cooking were also found.

We found out that the local outdoor levels of PM_2.5_, PM_2.5abs_, and ozone were the most important determinants of the personal and indoor levels of the same air pollutants in the studied suburban residential area. Particles and gases penetrate from outdoor air to indoors. Besides outdoor air concentration of pollutants, building tightness and ventilation system, and resident's behavior, such as keeping a window open, affect the infiltration of fine particles and gases indoors.^7^, ^11^, ^33^ In the northern subarctic climate, building envelopes are tightly sealed to prevent heat leaks, which also decreases the infiltration of particles, especially fine particles. However, infiltration is still a significant factor for indoor concentration. The mean infiltration factor for PM_2.5_ was 0.59 in Helsinki, the capital city of Finland, in a study conducted in four European cities in winter and spring 1998‐1999.^34^ Infiltration factors were higher in the other three cities, which were located in central and southern Europe.

Building air exchange rate is dependent on the ventilation system. Fully mechanical ventilation systems that most often have coarse and fine PM filters in the supply air side of the ventilation unit are typically more effective than natural ventilation, not only with regard to the air exchange rate but also in the reduction of PM infiltration indoors.^33^ In this study, natural ventilation was associated with 21% higher personal PM_2.5_ levels compared to at least partly mechanical ventilation system. This may be, at least partly, a result from the accumulation of particles from indoor sources in the home indoor air instead of effective removal outdoors. However, the same association was not found between ventilation and PM_2.5abs_. In the studied detached and row houses, mechanical exhaust ventilation systems were more common than mechanical supply ventilation.

Spearman correlations between central outdoor and indoor PM_2.5_ concentrations in this study were relatively low (median *R* = 0.4). This supports the idea of the relative importance of indoor sources, but it may also be related to the generally low range of the concentrations or concentration fluctuation due to local sources. Janssen et al.^35^ have studied longitudinal Pearson's correlations between central outdoor, indoor, and personal exposure levels of PM_2.5_ and PM_2.5abs_ in Helsinki and in Amsterdam, the Netherlands. Individual level correlations between indoor and central outdoor environments were high for PM_2.5_ (median *R* = 0.70 in Helsinki and 0.84 in Amsterdam) and even higher for PM_2.5abs_ (median *R* = 0.74 and 0.96) in both cities. Home outdoor concentrations have correlated in the range of 0.51‐0.82 with indoor concentrations in a three‐city study by Montagne et al.^36^ The lowest correlation was observed in Helsinki. Similar level correlations have also been found in the study of Hoek et al.^37^ Pearson's correlations between home outdoor and indoor levels of PM_2.5_ in Helsinki and Amsterdam were 0.74 and 0.85, whereas the correlations in Athens and Birmingham were lower (0.63 and 0.35, respectively).

In our study, the local central outdoor ozone concentration was a determinant of indoor ozone level. Earlier studies have reported a strong link between indoor and outdoor ozone concentrations.^11^ According to Fadeyi et al,^38^ ozone concentrations in indoor environments are generally low, but they may be strongly influenced by the use of certain types of room air‐cleaning devices, some of which generate considerable amounts of ozone. Photocopiers and laser printers may also produce ozone.^11^ Only five subjects in our study used a room air cleaner, and only on eight days in total. Thus, the influence of these particular devices on indoor ozone concentration was minor.

Candle burning has been recognized as a major indoor particle source.^26^, ^29^ In this study, candle burning affected both personal and indoor PM_2.5abs_ levels. Candle burning increased personal PM_2.5abs_ levels 8% and indoor PM_2.5abs_ 10%. Interestingly, candle burning was also associated with a slightly higher (3%) home indoor ozone concentration in this study. However, we have no obvious explanation for this finding, which also may have appeared merely by chance. In one previous study, a negative association between candle burning and indoor ozone has also been reported.^39^

Wood burning was associated with higher levels of personal and indoor PM_2.5abs_, and it also increased personal PM_2.5_ levels. During those measurement days that wood burning took place, it lasted on average 2.3 hours. This led to 9% and 20% increases in personal PM_2.5abs_ and PM_2.5_ exposure levels, respectively, and a 7% increase in the indoor PM_2.5abs_ level. We did not find any association between wood burning and daily levels of indoor PM_2.5_. A clear association between the use of a fireplace and indoor particle concentrations in winter time has been found in a study conducted in Italy in southern Europe.^40^ One important difference between this study and our study is the type of room heaters used by people in different countries. In Finland, closed masonry heaters with glass doors or other types of closed stoves or ovens are the most popular installations. Unlike open fireplaces, these heater types have high burning efficiency and the relatively short duration of wood burning generates heat indoors from the stone structure for 1‐2 days. Moreover, they emit much less flue gas indoors than open fireplaces during wood burning.

Cooking with electric stoves and/or ovens was associated with increased levels of indoor PM_2.5_. However, no association was found between cooking and personal exposure to PM_2.5_ or PM_2.5abs_. This cannot be explained by cooking activities conducted by other residents, as 46% of cooking activities were conducted by study subjects themselves. Time spent in cooking also seemed to be slightly longer when cooking was done by the study subject themselves. In the study by Lanki et al,^25^ cooking was a significant determinant of both personal and indoor PM_2.5_ and PM_2.5abs_, while PM_abs_ was not associated with cooking in the present study. There is no obvious explanation for the contradiction in the PM_2.5abs_ results. It can be hypothesized that wood and candle burning may overpower the effect of cooking on certain air pollution parameters in home indoors. In earlier studies, cooking activities have also been associated with ultrafine particles,^29^ PM_0.02‐0.5_ and PM_0.7‐10_ in indoor air.^28^

In contrast to previous studies,^25^, ^40^ we did not find any association with traffic density (collector road within 50 m) or time spent in a vehicle with the personal or indoor PM_2.5_ and PM_2.5abs_ levels. This is likely due to a relatively small amount of traffic in Jynkkä, where the mean concentrations of traffic emissions and road dust have been approximated to be 0.7 and 0.6 µg/m^3^, respectively.^19^

Wood combustion is a significant source of ambient air pollution in northern countries during the winter season. In Finland, district heating is common in the cities, but small‐scale wood combustion as a secondary heating source and wood‐fired sauna stoves are widely used in residential areas. In the study area, the mean outdoor concentration of PM_2.5_ from wood burning was 0.9 µg/m^3^ on the basis of a previous source apportionment.^19^ However, the central site outdoor level of PM_2.5_ from wood combustion was not associated with personal or indoor levels of any PM parameter. This suggests that data from a local central monitoring site may accurately represent only the actual exposure levels of those residents who live in the immediate vicinity.^19^

Winter 2008‐2009 and spring 2009 were warmer than usual in Finland, which most probably decreased wood combustion for secondary heating. This may have resulted in lower than usual exposure to wood combustion related air pollutants. However, there was a wood‐fired sauna stove in ten residences out of 37, and these stoves were used, on average, 2.4 times per week regardless of the outdoor temperature. In any case, milder winters are becoming more common in future because of the climate change. The average wintertime temperature (November‐March) has increased 1.1°C from January 1981‐March 2008 to November 2008‐March 2018 in Kuopio. One limitation of the study is the relatively small number of repetitions of 22‐hour measurements. Another limitation is the lack of information on air exchange rates. Further, study subjects were elderly persons and almost all of them were retired. Therefore, results may not be generalizable for other age groups, such as school children or the working age population, who spend a notable part of the day outside the home.

Smoking indoors is a dominant source of the PM_2.5_ and PM_2.5abs_ in indoor air, if anyone is smoking indoors.^25^, ^26^, ^41^ A strength of this study was the lack of smokers, allowing the estimation of other, more sparsely studied indoor emission sources. Moreover, measurements of both personal and indoor levels of the selected pollutants could be done. Finally, the low level of regional outdoor air pollution enhanced our changes to identify and quantify individual indoor sources, as the absolute amount of particles in outdoor air did not strongly dominate personal exposure and home indoor concentrations.

## 5 CONCLUSIONS

Local outdoor air concentrations of PM_2.5_, PM_2.5abs_, and ozone were important determinants of their daily home indoor and personal exposure levels in a suburb of the northern European city of Kuopio, Finland, which has a generally low level of air pollution. Natural ventilation of the detached and row houses increased total personal exposure to PM_2.5_, but not to PM_2.5abs_, when compared to mechanical ventilation. Only cooking out of the recorded household activities increased indoor PM_2.5_. The use of a wood stove room heater or wood‐fired sauna stove was associated with elevated concentrations of personal PM_2.5_ and PM_2.5abs_, and indoor PM_2.5abs_. Wood and candle burning were significant predictors of residents’ higher indoor and personal exposures to potentially hazardous, combustion‐derived carbonaceous particulate matter.

## CONFLICT OF INTEREST

The authors have no conflict of interest to declare.
